# Is there a Function for a Sex Pheromone Precursor? A Predicted Link between Bacterial Redox Metabolism and Propagation of Antibiotic Resistance

**DOI:** 10.1515/jib-2019-0016

**Published:** 2019-07-13

**Authors:** O. Vasieva, I. Goryanin

**Affiliations:** University of Liverpool, Crown street, Liverpool, UK; Ingenet ltd, 3d floor, 207 Regent street, London, UK; University of Edinburgh, Edinburgh, UK; Okinawa Institute Science and Technology, Okinawa, Japan; Tianjin Institute of Industrial Biotechnology, Tianjin, China

**Keywords:** ApbE, cAD1, *Enterococcus*, redox metabolism, SEED

## Abstract

Functional coupling and comparative genomics analysis have been applied to study functional associations of orthologs of enterococcal cAD1 sex pheromone (P13268) known to be responsible for biofilm formation, conjugative plasmid transfer and spreading of bacterial antibiotics resistance. cAD1 peptide pheromone is released from the membrane lipoprotein with the peptide precursor encoded by a gene *cad* (tr|C2JQE7). Our analysis of genomic neighbourhood of *cad* and motifs of the encoded polypeptide and its orthologs suggests a close functional association between cAD1 and ApbE protein (Q82Z24), a FMN insertion and trafficking facilitator. The *cad* and *apbE* orthologs were coupled in the genomes and ApbE-specific motifs for FMN covalent attachment were identified in *cad*-encoded protein sequence and its orthologs. These findings suggest a potential role of FMN-based reductase function of the cAD1 lipoprotein precursor in its processing and release of the active sex pheromone peptide. They may lead to a new approach in prevention of antibiotic resistance spread via targeting sex pheromone processing chaperones or by suppression of the FMN availability and covalent binding. This methods can be also applied to a controlled evolution of bacterial pathogenicity in microbial fuel cells, as the findings suggest the crosstalk between bacterial pathogenicity and bacterial electro-activity.

## 1 Introduction

Recently developed insights in microbial redox metabolism [1], [2], [3] and emerged biotechnology of microbial fuel cells (MFC) [4] provided new data enabling to link cell redox processes and electron transfer to functions involved in virulence and bacterial pathogenicity, previously considered as largely autonomous. Suppression of pathogenic traits, observed in MFC under an applied electric potential [5], [6] in some groups of bacteria, is the fact that leaves more questions than answers. Functional links between bacterial redox metabolism and factors triggering biofilm formation and conjugational processes are coming to the focus of the research, and this in silico study establishes one of the potential connections.

Functional coupling of genes in bacterial genomes was demonstrated on multiple examples and is used to predict protein functions and new pathway connections [7], [8] A number of tool has been developed to support this analytical approach [7] and are at the moment among the most cited in the area of bacterial genomics. We have applied this method to infer functional connections of cAD1 sex pheromone (P13268) [9] produced by *Enterococcus*. As other known bacterial sex pheromones, cAD1 is secreted by plasmid-free cells to be recognised by plasmid-baring cells and initiate cell clumping, conjugation and the virulence pAD1 plasmid transfer [10], [11], [12]. This processes are tightly associated with biofilm formation and a number of other pathogenic determinants and are triggered by stressors, especially in a form of free radicals [13], [14], [15].

Conjugative transfer is the most efficient way of horizontal gene spread, and it is therefore considered one of the major reasons for the increase in the number of bacteria exhibiting multiple-antibiotic resistance [16]. Thus, conjugation and spread of antibiotic resistance represents a severe problem in antibiotic treatment, especially of immunosuppressed patients and in intensive care units. No consistent data exist on regulation of the pheromone peptide signalling by mechanisms associated with the metabolic state of a cell, its potential triggers and facilitators.

The known enterococcal sex pheromones (cAD1, cPD1, cCF10, cAM373, and cOB1) [15], [17] are all relatively hydrophobic, linear octa- or heptapeptides that are active at nanomolar concentrations. They all are proteolytically processed from the precursors-longer membrane-bound proteins with non- identified functions in different bacteria, as well as in fungi [18], [19]. Certain amino-acid motifs are conserved in pheromone lipoproteins within at least one orthology class [20].

## 2 Workflow

Our analysis of motifs of cAD1 sex pheromone precursor protein and its orthologs, as well as the genomic neighbourhoods of *cad* homologs in different genomes suggests close functional association between cAD1 and ApbE protein (Q82Z24), known to be involved in thiamine metabolism in *Enterobacteria* [21], recently associated with flavin trafficking in *Treponema* [22] and confirmed to be a FAD/FMN insertion chaperon [23], [24]. This suggests a potential role of FMN availability or its redox-dependent trafficking in the sex pheromone precursor processing and, consequently, biofilm formation, bacterial pathogenicity and antibiotic resistance.

**Figure 1:**
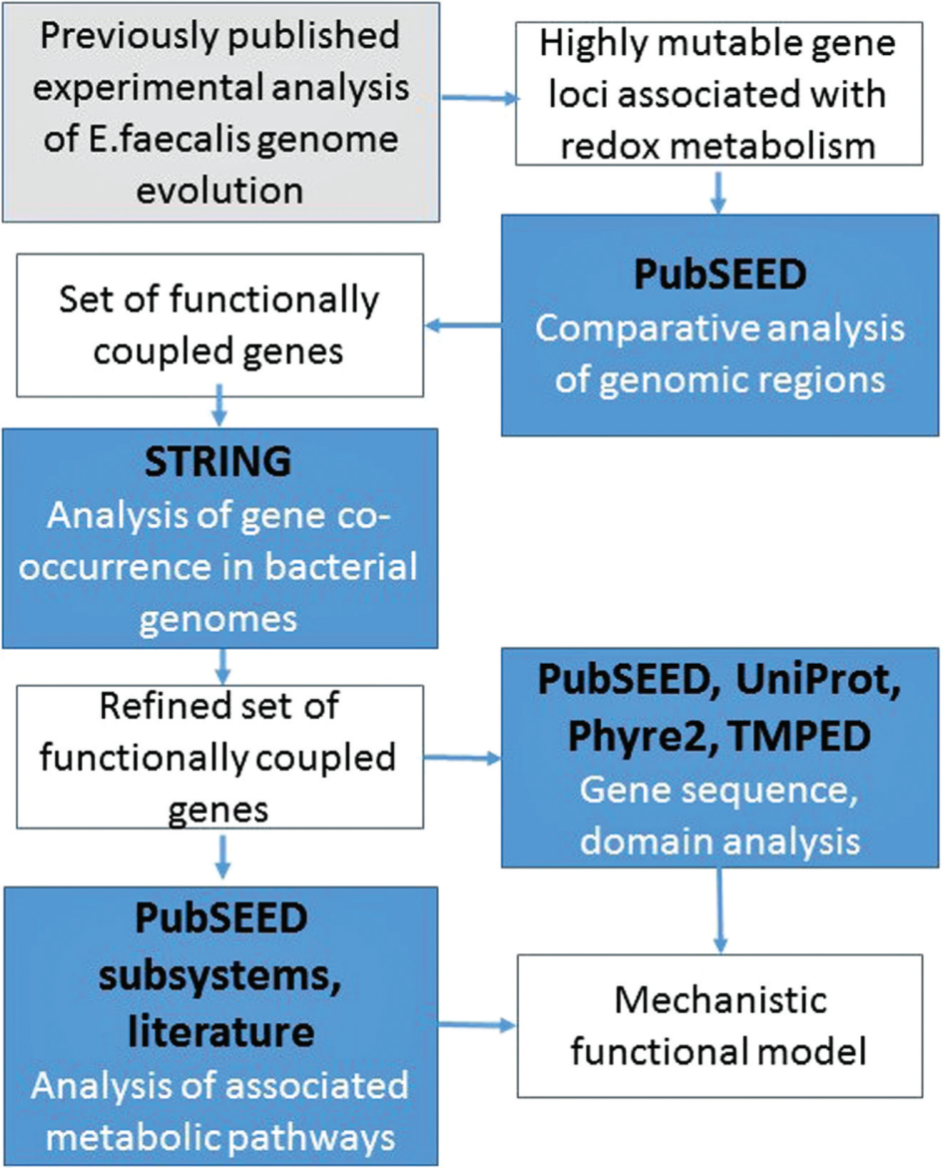
Illustration of the applied work flow. SEED platform (http://pubseed.theseed.org/) was used to retrieve and compare the bacterial genomic regions associated with genes of interest. Analysis of genes co-occurrence in the genomes and their connectivity were performed in STRING (https://string-db.org/) environment. SEED, STRING and Uniprot (https://www.uniprot.org/) tools were applied to analyse protein structural features and the relevant metabolic pathways.

A workflow for the presented analysis is depicted in Figure 1. SEED Viewer (PubSEED) and the associated comparative genomics platform (http://pubseed.theseed.org) have been used in this study. The SEED provides precise genome annotations that are cross-validated and unified across the genomes. It is a platform for a comparative functional and phylogenetic analysis of multiple sets of genomes and the basis for RAST annotation service. [7]. It also integrates information from validated sources (Kegg, NCBI, ProDom, etc) and manual curation, and can be explored in terms of sequences, pathways and functional subsystems analysis. The basic information and genomic regions containing homologs of cAD1 precursor encoding gene (UniProt: P13268) (SEED ID: fig|226185.9.peg.3033) were retrieved for the comparative analysis. ‘Compare region’ application has been used for the homologous bacterial genomic regions retrieval (set e-20 similarity threshold) and structural comparison. ‘Compare regions’ diagrams present homologous genes as same color-coded arrows with the same associated number (a direction of an arrow indicates the coding vector). The regions are aligned (pinned) via the seed gene homologs (presented as N1, red in all the diagrams).

STRING (http://string-db.org) analytical platform has been used to reconstruct functional associations between genes in the studied genomic regions by integration of known and predicted protein interactions [25]. The input proteins are presented graphically in the context of their interaction networks and phylogenetically associated patterns of the genomic neighbourhoods, gene co-occurrence and the expression profiles. Multiple protein names are used as an input and are mapped to the database organisms/genes via textual associations.

The input ‘FAD:protein FMN transferase’ and ‘pheromone *cad1*’ has been associated with *Enterococcus faecalis* among other organisms, and the following genes were chosen for the STRING analysis: EF_1225 – Thiamine biosynthesis ApbE; Flavin transferase that catalyzes the transfer of the FMN moiety of FAD and its covalent binding to the hydroxyl group of a threonine residue in a target flavoprotein (a.k.a. EF1225, NP_814952.1, 1200125, FAD:protein FMN transferase), EF_3256 – Pheromone cAD1 lipoprotein (a.k.a. NP_816853.1, EF3256, Q82Z23_ENTFA). The network was expanded by one more shell to automatically include the genes in the input genes genomic proximity: EF_3254-1,4-dihydroxy-2-naphtoate octoprenyltransferase and EF_3257 –Pyridine nucleotide-disulphide family oxidoreductase. These genes were selected in the reconstructed network and automatically added to the input list of functions.

‘FAD:protein FMN transferase’ and ‘Electron transport complex rnf’ input has been associated with *Clostridium genus* among other organisms, and the genes listed below were chosen for the following analysis: CD2483 – Hypothetical protein; Flavin transferase that catalyzes the transfer of the FMN moiety of FAD and its covalent binding to the hydroxyl group of a threonine residue in a target flavoprotein (a.k.a. CD630_24830, YP_001088997.1, AM180355, FAD:protein FMN transferase), *rnfC* – Electron transport complex protein (a.k.a. CD630_11370, YP_001087628.1, CD1137, Electron transport complex protein), *rnfD* – Electron transport complex protein (a.k.a. CD630_11380, YP_001087629.1, CD1138, Electron transport complex protein), *rnfG* – Electron transport complex protein (a.k.a. CD630_11390, YP_001087630.1, CD1139, Electron transport complex protein), *rnfE* – Electron transport complex protein (a.k.a. CD630_11400, YP_001087631.1, CD1140, Electron transport complex protein), *rnfA* – Electron transport complex protein (a.k.a. CD630_11410, YP_001087632.1, CD1141, Electron transport complex protein), *rnfB* – Electron transport complex protein (a.k.a. CD630_11420, CD1142, YP_001087633.1, Electron transport complex protein).

The ‘neighbourhood’ and ‘co-occurrence’ STRING views were chosen for graphical outputs of the associations between the input genes. For automated co-occurrence profiles STRING uses a phylogenetic profiling algorithm, SVD-Phy which performs truncated singular value decomposition to address the problem of uninformative profiles giving rise to false positive predictions. The graph shows all scores co-occurrences detected.

Phyre2 (Protein Homology/analogY Recognition Engine V 2.0 analysis, http://www.sbg.bio.ic.ac.uk/phyre2) was applied to reconstruct potential tertiary structure of cAD1. A crystal structure of cpe2226 protein from* Clostridium perfringens*, annotated in SEED as Putative pheromone precursor lipoprotein (fig|195102.1.peg.2289), and submitted by northeast structural genomics consortium (target cpr195) has been suggested by a search engine and used as a template for the 3D reconstruction with 99.9% confidence and 44% similarity to our query.

TMPRED (https://embnet.vital-it.ch/software/TMPRED_form.html) has been used for membrane-spanning motif prediction.

## 3 Application

### 3.1 cAD1 – ApbE Functional Coupling

**Figure 2:**
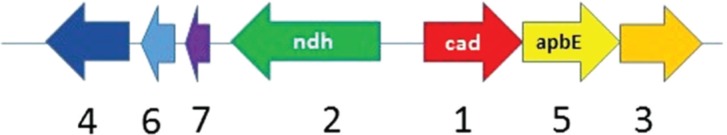
The *Enterococcus faecalis* gene locus containing cAD1 precursor (P13268) gene cad (tr|C2JQE7, fig|226185.9.peg.3033). 7500 bp region is shown. The arrows represent genes encoding for: 1- Pheromone cAD1 precursor lipoprotein Cad, 2- NADH dehydrogenase (EC 1.6.99.3), 3-1,4-dihydroxy-2-naphthoate polyprenyltransferase (EC 2.5.1.74),4- Heptaprenyl diphosphate synthase component I (EC 2.5.1.30), 5- Hypothetical similar to thiamine biosynthesis lipoprotein ApbE/FAD:protein FMN transferase (EC 2.7.1.180), 6- Heptaprenyl diphosphate synthase component I (EC 2.5.1.30), 7-hypothetical protein.

The *Enterococcus faecalis* gene locus containing cAD1 precursor (P13268) gene *cad* (tr|C2JQE7) represents an interesting combination of links to virulent and metabolic functions (Figure 2, Table 1). The two genes that are in an immediate proximity to *cad* encode for enzymes involved in electron transfer process or its modulation, and 3 other genes are relevant to quinone biosynthesis.

**Table 1:** Annotations and associated IDs for genes in cAD1 precursor/cad locus in *Enterococcus faecalis* V58 genome.

*n*	ID	Feature in SEED	bp	Function	FigFams
1	tr|C2JQE7	fig|226185.9.peg.3033	930	Pheromone cAD1 precursor lipoprotein Cad	FIG00132896: Pheromone cAD1 precursor lipoprotein Cad
5	tr|C0X0V8	fig|226185.9.peg.3032	1068	FAD:protein FMN transferase (EC 2.7.1.180) @ FAD:protein FMN transferase (EC 2.7.1.180), HepST-associated	FIG00132666: Hypothetical similar to thiamin biosynthesis lipoprotein ApbE
2	tr|C0X0V5	fig|226185.9.peg.3034	1947	NADH dehydrogenase (EC 1.6.99.3) in cluster with putative pheromone precursor	FIG01304395: NADH dehydrogenase (EC 1.6.99.3) in cluster with putative pheromone precursor
3	tr|C2JQE5	fig|226185.9.peg.3031	948	1,4-dihydroxy-2-naphthoate polyprenyltransferase (EC 2.5.1.74)	FIG00085608: 1,4-dihydroxy-2-naphthoate polyprenyltransferase (EC 2.5.1.74)
6	tr|C0X0V3	fig|226185.9.peg.3036	558	Heptaprenyl diphosphate synthase component I (EC 2.5.1.30)	FIG00004229: Heptaprenyl diphosphate synthase component I (EC 2.5.1.30)
4	tr|Q82Z19	fig|226185.9.peg.3037	987	Heptaprenyl diphosphate synthase component II (EC 2.5.1.30)	FIG00002487: Heptaprenyl diphosphate synthase component II (EC 2.5.1.30)

‘Feature in SEED’ corresponds to an ID in SEED database, *n*- number of a gene associated with Figure 1 genomic regions, aa-protein length, FigFams-protein family ID and its annotation in SEED database.

**Figure 3:**
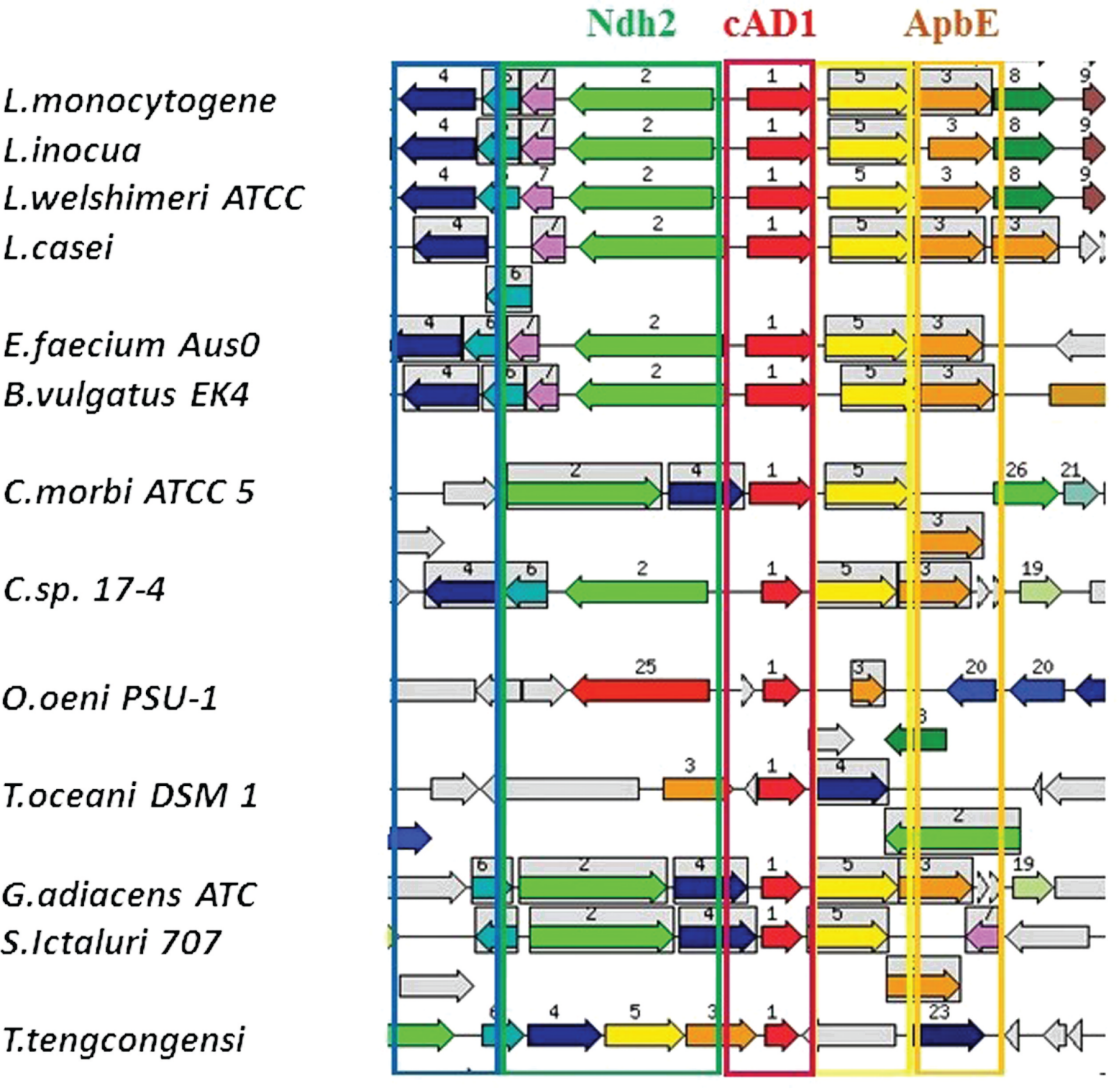
The genomic regions containing cAD1 precursor encoding cad gene and its close homologs retrieved from SEED database. Seed gene: fig|226185.9.peg.3033; e-20 similarity thresholds set for region retrieval and color-coding of the homologs. Regions shown correspond to 16,000 bp window. The arrows represent genes, same colour and number depicts homology, frames surround groups of homologous genes. Grey arrows represent genes that do not have homologs in the shown regions. The genes and encoded proteins are annotated in SEED as following: Red N1 – cad, Pheromone cAD1 precursor lipoprotein Cad; yellow N5-apbE, FAD:protein FMN transferase (EC 2.7.1.180); green, N2- NADH dehydrogenase (EC 1.6.99.3); orange, N3- 1,4-dihydroxy-2-naphtoate polyprenyltransferase (EC 2.5.1.74), N4- Heptaprenyl diphosphate synthase component II (EC 2.5.1.30). The other numbers define orthologous genes with no conserved genomic coupling to the cAD1 and functions not obviously related to the discussed metabolic area. Abbreviated species of bacteria from top to bottom (the left panel): Listeria (top 5 species), Lactobacillus casei, Enterococcus faecium, Bacteroides vulgatus, Catonella morbi, Oenococcus oeni, Thermosediminibacter oceani, Granulicatella adiacens, Streptococcus ictaluri, Thermoanaerobacter tengcongensis.

**Figure 4:**
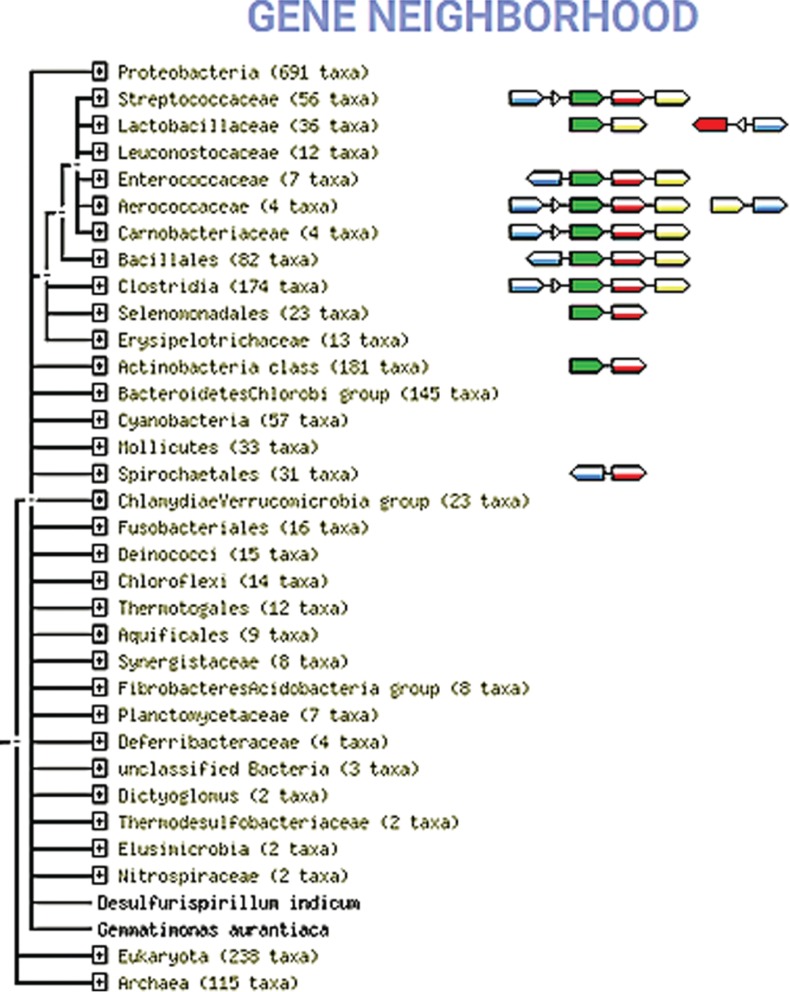
Co-occurrence and genomic proximity of genes encoding for homologs of cAD1 precursor (ref: Q82Z23, EF3256) and FAD: protein FMN transferase ApbE (ref: Q82Z24, EF1225). String database, gene neighbourhood view. cAD1 orthologs are depicted as green arrows, 1,4-dihydroxy- 2-naphtoate octoprenyltransferase (EF3254) –yellow arrows, and pyridine nucleotide-disulphide family oxidoreductase (EF3257)-blue arrows. The taxonomic tree used to display gene co-occurrence profiles was automatically generated by STRING and is based on canonical classification of all organism recorded in STRING database. The number of included taxa of low rank is shown in the brackets near each listed group name. STRING evaluates the phylogenetic distribution of orthologs of all proteins in a given organism. If two proteins show a high similarity in this distribution, i.e. if their orthologs tend to be observed as ‘present’ or ‘absent’ in the same subsets of organisms, then an association score is assigned [26].

**Figure 5:**
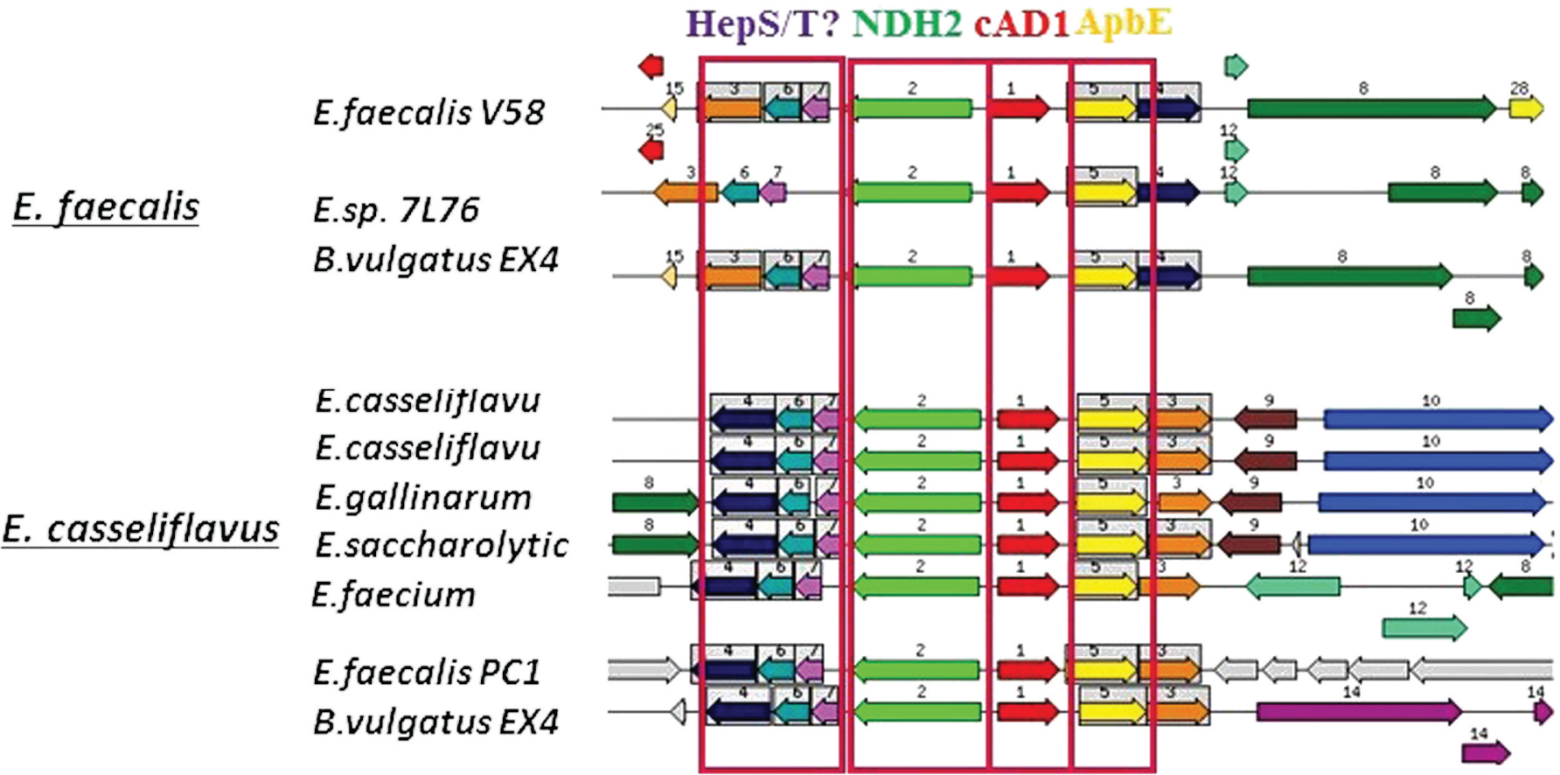
The genomic regions containing cAD1 precursor encoding cad genes from different *Enterococci* species (indicated at the beginning of each row) retrieved from SEED database. The seed input gene: fig|226185.9.peg.3033; e-20 similarity thresholds set for region retrieval and color-coding of the homologs. The arrows represent genes. Same frame and arrow colour and a number depict homologs. Regions shown correspond to 16,000 bp window. Grey arrows represent genes that do not have homologs in the shown regions. Two types of the adjacent neighbourhoods are associated with the representative Enterococcus species (the left panel). Red N1 – cad, Pheromone cAD1 precursor lipoprotein Cad; yellow N5-apbE, FAD:protein FMN transferase (EC 2.7.1.180); green, N2- NADH dehydrogenase (EC 1.6.99.3); purple, N7-HepS/T-heptaprenyl diphosphate synthase component, Red N1 – cad, Pheromone cAD1 precursor lipoprotein Cad; yellow N5-apbE, FAD:protein FMN transferase (EC 2.7.1.180); green, N2- NADH dehydrogenase (EC 1.6.99.3); N4- Heptaprenyl diphosphate synthase component II (EC 2.5.1.30). The other numbers define orthologous genes with no conserved genomic coupling to the cAD1 and functions not obviously related to the discussed metabolic area.

The genomic regions from different bacteria containing close homologs to cAD1 precursor (P13268) gene *cad* (Table 1, tr|C2JQE7) were retrieved from SEED database by means of ‘Compare region’ application with e^−20^ similarity thresholds set for region retrieval and color-coding of the homologs (Figure 3). The most striking information derived from this analysis was a strong positional association, with almost no exceptions between *cad* homologs and *apbE* homologs encoding thiamine biosynthetic protein ApbE/FAD:protein FMN transferase (Q82Z24) (Table 1, Figure 2–Figure 4). The conservative genomic regions (Table 1, Figure 2) also contained genes for: NADH-dehydrogenase 2 (N2, green arrows) as well as prenyltransferases and heptaprenyl diphosphate synthase subunits, representing elements of isoprenoid biosynthetic pathway (N 3, 4, 6-orange, blue, turquois arrows). The 1–6 module was conserved across the heterogeneous group of bacteria including Gram positive (diverse representatives of class Bacili (*Lactobacillus, Listeria, Oenococus* genera) and Clostridia, and Gram negative (classes of Clostridia (*Catonella*), Bacteroidetes (*Bacteroides*), (Spirochaete) representatives (Figure 3 and Figure 4). The core cluster was very conservative in all the *Enterococci* species (Figure 2 and Figure 5). However, 2 types of adjacent gene clusters were noticed (Figure 5). Only *Enterococcus casseliflavus* type, also including *Enterococcus faecium*, contain gene encoding for glutathione reductase (tr|C0X0U2) among the other differences in the immediate cluster proximity.

### 3.2 Coupling between ApbE and Redox Functions

**Figure 6:**
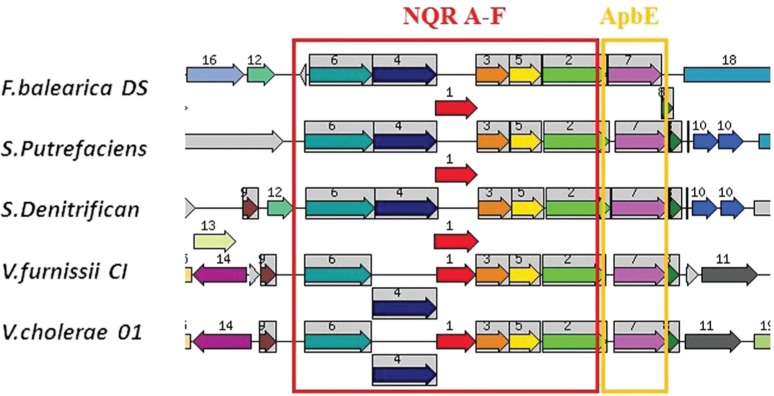
Example of genomic regions containing nqr A-F gene cluster and apbE orthologs retrieved from SEED database. Seed gene: nqrC, *Ferrimonas balearica* DSM (9799fig|550540.3.peg.901); e-20 similarity thresholds set for region retrieval and color-coding of the homologs. The arrows represent genes, same arrow colour and a number depict homologs. Grey arrows represent genes that do not have homologs in the shown regions. Regions shown correspond to 16,000 bp window. The genes and encoded proteins are annotated in SEED as following: N1-6, framed,- Nqr A-F, Na(+)-translocating NADH-quinone reductase subunit (A-F) (EC 1.6.5.-), N7-apbE homologs- FAD:protein FMN transferase (EC 2.7.1.180) @ FAD:protein FMN transferase (EC 2.7.1.180), NqrBC-associated; Abbreviated species of bacteria from top to bottom (the left panel): *Ferrimonas balearica* DSM 9799, 2, 3-Shewanella species, 4,5-Vibrio species.

Analysis of functional coupling of *apbE* homologous genes via application of STRING [25] and SEED [7] have shown they consistently associated with *nqr*(*A-F*) gene cluster in Gram negative bacteria possessing the encoded Na+ transporting NADH-quinone oxidoreductase, such as Vibrionaceae (*Vibrio, Shewanella, Ferrimonas*) (Figure 6), Moraxellaceae (*Psychrobacter*). Interestingly, most of these genome have duplicated nqr clusters, and only one of them associated with *apbE* homolog.

*ApbE* homologs are also frequently associated with *rnf* gene clusters, which is especially characteristic for Rnf-complex expressing Firmicutes, Actinobacteria, Bacteroidetes, Mollicutes and some representative of Fusobacteria (*Sebaldella*). A distant homology between cAD1 precursor and FMN-binding subunit of electron transfer Rnf complex, RnfG, has been mentioned [27], [28], [29] and suggests the evolutionary and/or functional link between RnfG and cAD1 precursor. In support of that, *cad*-*apbE* genomic associations appear only in genomes from which *rnf* genes are missing (Figure 4).

Though *nqr* loci do not contain other genes typical for *cad loci, rnf* gene clusters are also coupled to 3 genes involved in isoprenoid biosynthesis (encoding for biosynthetic function in nonmevalonate branch of isoprenoid biosynthesis and-heptaprenyl-dipohosphat synthase) and some redox functions (as genes encoding for NAD/FAD utilising hydrogenase). Such similarity suggests some degree of functional redundancy between cAD1 precursor and Rnf complex, which is also based on a commonality of their dependence on covalently attached FAD/FMN cofactor.

### 3.3 FMN Binding Motifs in cAD1 Precursor

FMN covalent-binding sites targeted by ApbE, such as in RnfG, share S(T)GAT amino acid motif, that is found to be required for ApbE chaperone activity [28]. They are different from the traditional non-covalent FMN binding sites existing in the majority of FMN-binding oxido-reductases [28]. We’ve also found single SGAT motifs in all annotated NqrC proteins, independently on their genomic coupling to ApbE homologs.

**Figure 7:**
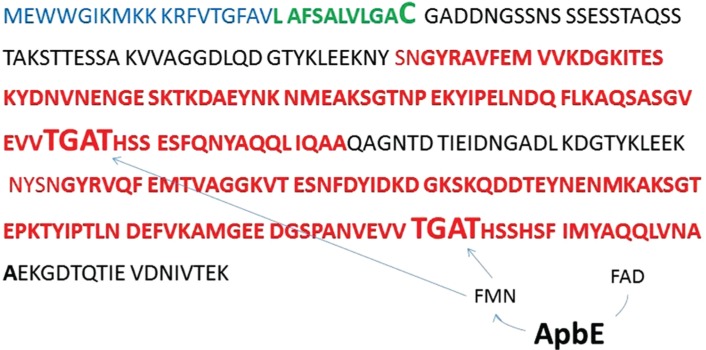
ApbE specific binding motifs for FMN insertion in cAD1 (P13268) sex pheromone protein precursor (SEED ID: fig|226185.9.peg.3033). The ApbE-dependent FMN binding sites are shown in bold, in red-the homologous sequences comprising the catalytic domain. The cAD1 peptide is shown in green. A signal sequence in shown in blue.

We used this motif to search for ApbE-lead FMN binding sites in cAD1 precursor. Two sites of FMN binding were found in two homologous domains of cAD1 precursor protein (Figure 7).

All the analysed protein sequences of cAD1 precursor orthologs from other bacteria had one or two SGAT motifs. For instance, two SGAT motifs are found in ortholohous *Listerial* pheromone protein precursors (ref (SEED): fig|386043.6.peg.2514 Pheromone cAD1 precursor lipoprotein Cad (*Listeria welshimeri serovar 6b str. SLCC5334*)), *Lactobacillus* (ref (SEED): fig|525337.3.peg.1495 Pheromone cAD1 precursor lipoprotein Cad (*Lactobacillus paracasei subsp. paracasei ATCC 25302*)) and one SGAT motif is detected in *Oenococcal* ortholog (ref (SEED): fig|203123.5.peg.1084 Pheromone cAD1 precursor lipoprotein Cad *Oenococcus oeni PSU-1*).

Similar, TGAS and TGAV motifs are identified in succinate dehydrogenase subunits, where the first motif is also associated with covalent FAD/FMN binding [24]. ApbE may potentially have a central function in metabolism of the discussed bacteria enabling specific covalent flavin-based electron transfer and, potentially, flavin trafficking.

**Figure 8:**
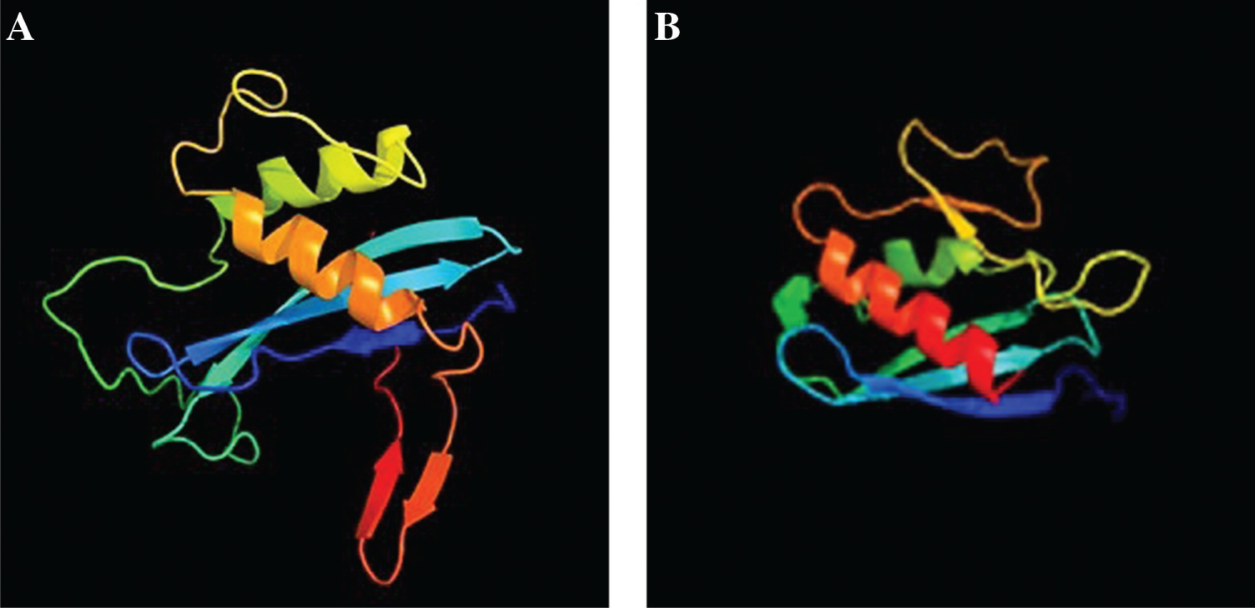
Results of comparison of the reconstructed tertiary structure of cAD1 to the library of known 3D protein structures via Phyre2 (Protein Homology/analogY Recognition Engine V 2.0 analysis). A crystal structure of cpe2226 protein from *Clostridium perfringens*, cAD1 homolog annotated in SEED as Putative pheromone precursor lipoprotein (fig|195102.1.peg.2289) was used as a template for the 3D reconstruction (44% similarity to the query). 117 residues (38% of the sequence) have been modelled with 99.9% confidence by the single highest scoring template. A-reconstructed tertiary structure of cAD1, Model dimensions (Å):X:33.109 Y:47.972 Z:39.437; B-3D reconstruction of NqrC subunit of Na+ translocating NADH-quinone reductase based on crystal structure of Shewanella oneidensis NqrC. Models are colored by rainbow from N to C terminus.

Phyre2 (Protein Homology/analogY Recognition Engine V 2.0 analysis) has been applied to reconstruct potential tertiary structure of cAD1. A crystal structure of cpe2226 protein from *Clostridium perfringens*, annotated in SEED as Putative pheromone precursor lipoprotein (fig|195102.1.peg.2289), and submitted by northeast structural genomics consortium (target cpr195) has been suggested by a search engine and used as a template for the 3D reconstruction with 99.9% confidence and 44% similarity to our query. Interestingly, the closest functionally annotated match (98% confidence and 17% similarity with cAD1 within 56–163 residues) was Na+-translocating NADH-quinone reductase subunit C (*nqrc*) from *Shewanella and Vibrio Cholera* (Figure 8).

Tertiary structure of cAD1 125–291 residue part was also found to have 11% similarity to Na+-translocating Nadh-quinone reductase 2 subunit C from *Parabacteroides distasonis* (yp_001302508.1). cAD1 possesses one transmembrane motif between 6–20 amino acids, identified by TMPRED program in cAD1 with a significant score of 1573.

Detailed and integrative structural analysis of the full polypeptide will be required for more meaningful functional prediction, however, as seen from the Figure 8 cAD1 and Na+ translocating NADH-quinone reductase subunit C share certain structural features.

## 4 Discussion

Unlike the majority of oxidoreductases that contain non-covalently associated FMN molecule, some redox-related enzymes contain covalently-bound cofactor, which receives electrons from NADH, and a tightly bound ubiquinone that mediates electron transfer from FAD to the diffusible quinone pool [30]. It has been recently shown that the ApbE thiamine biosynthetic protein homolog [21] is required for an attachment of an FMN residue to proteins via a phosphoester bond [23], [24], [31] and is also involved in extracellular flavin trafficking [22]. RnfG and NqrC subunits of the redox complexes, examples of covalently flavinated proteins [22], [24], [29], possess an ApbE-specific FMN insertion motif (TGAT) [24], [28] and are functionally coupled to ApbE homologs (Figure 6).

**Figure 9:**
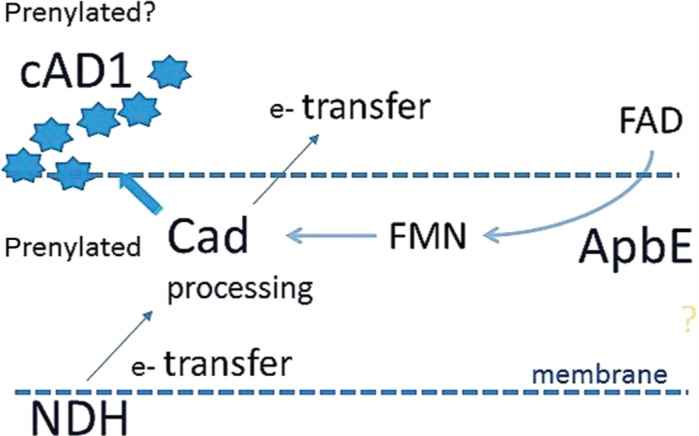
Suggested functional interaction between cAD1 pheromone and ApbE/FAD:protein FMN transferase. The release of cAD1 peptide pheromone may depend on a FMN-based electron transfer activity or a conformation of the lipoprotein precursor (Cad). ApbE works as a chaperone facilitating covalent binding of FMN and FAD/FMN extracellular trafficking.

Enterococcal sex-pheromone lipoprotein precursor, cAD1, also occurred to be a FAD/FMN-binding protein. According to our analysis the cAD1 precursor and its orthologs from other bacteria are encoded by genes adjacent to *apbE* homologs (Figure 2–Figure 5). A number of mentioned above Firmicutes genera [32], [33] and particularly *Listeria* [34], *Catonella* [35] representatives, as well as Bacteroidia (*Bacteroides*) [36], were considered as electrogenic and shown to populate MFCs [4] under different conditions. We show that cAD1 precursor has two sites specific for ApbE-dependent FMN insertion (TGAT) (Figure 7), and its homologs have at least one FMN covalent binding insertion motif. Structurally, cAD1 shares features typical for NqrC subunit of Na+ translocating NADH-quinone reductase (Figure 8) and possesses one transmembrane motif. In electroactive bacteria these proteins may comprise a part of a flavin trafficking [22], [37], external electron transfer cycle (Figure 9) and, hypothetically, serve a signalling function. Availability of extracellular flavins, as a measure of a communal density and of it’s integral metabolic potential, may be translated into cAD1 precursor activity and has effect on its accessibility for proteases.

A number of studies demonstrated an importance of flavin trafficking for electrogenic metabolism in bacteria [38]. From our analysis and the information discussed above, it would be logical to suggest that the particular sex pheromone systems can be also directly linked to the redox regulation and bacterial electroactivity. Interestingly, many bacteria that possess Nqr or Rnf complexes or cAD1 homologs are known as electrogens. Presence of these clusters and, particular, *cad-apbE* gene cluster in a genome may be used as a predictive measure. There are still no reports for electroactivity of *Oenococcus* genera but, based our analysis, for instance, we can suggest that this genus also has electrogenic properties. We may even suggest, taking in mind pheromone properties of cAD1, that its processing can be one of regulators of relevant bacteria response to an applied electric potential and, for these bacteria groups, a trigger of the anodic biofilm formation in MFC [37], [39].

The analysis of the *cad* gene functional coupling suggests that prenylation of the terminal cysteine [40] is also involved in the processing of cAD1 peptide and in the electron transfer function of the pheromone precursor [30], which can be taken in account in development of new drugs supressing propagation of antibiotic resistance, for instance, based on inhibitors of isoprenoid biosynthesis. *Nqr* genes, typical for rather Gram negative bacteria possessing periplasm, were not found to be functionally coupled to metabolism of isoprenoids. It suggests that potential prenylation function associated with Rnf or cAD1 orthologs may reflect a need in membrane anchoring of these or other components of the protein complexes on the membrane.

Ironically, while we were preparing our manuscript, the publication in Nature appeared [41] that experimentally proved a link of *Listeria* pheromone PlpA (homologous to cAD1 according to our analysis) to the electron transfer and favin trafficking. This striking coincident indicates the timely significance of the described phenomenon, and we see two studies as pretty much complementary. Our enquiry has been commenced after the earlier research [42] where analysis of an advantage of *Enterococcus faecalis* over *Staphylococcus aureus* in a mixed host residential population pointed to an importance of the *cad* locus. In this work [42], NADH dehydrogenase gene (tr|C0X0V5, N2 in Table 1) was shown to be among the most frequently mutated in *E. faecalis* genome under the mixed population experimental conditions. These data support the involvement of cAD1 precursor locus in overall bacterial pathogenicity, competition and resistance.

A relation of the suggested electron transfer function of cAD1 precursor to spreading of antibiotic resistance in natural bacterial populations would be of a medical and biotechnological importance. It is especially intriguing, due to an extreme virulence of bacteria baring this sex pheromone system (among which are *Enterococcus, Streptococcus, Staphylococcus*) and documented spreading of antibiotic resistance in-between these species [43]. Similar to cAD1’s ones, active or even cryptic motifs may be also discovered in evolutionary related proteins which can constitute new drug targets in other pathogens.

Antibiotics to which the conjugative transfer in *Enterococcus* is especially sensitive, such as vancomycin [44] are macrolides whose antibacterial action is based on cell membrane potential disturbance [3], which supports a suggested relevance of sex pheromone release to redox sensing. We propose that FMN/FAD binding by a specific motif in a particular sex pheromone lipoprotein precursor may regulate its processing and a consequent release of the peptides. We claim that ApbE and the associated cofactors may be important regulators of conjugative transfer and biofilm formation in *Enterococcus, Staphylococcus, Streptococcus* and *Listeria* and can be explored for new approaches to stop propagation of antibiotic resistance in chronic diseases [1], [2], [3], [10], [11], [12], [13], [14], [16], [17], [18], [41], [42], [43], [45]. The method applied here also demonstrates a potential of *Insilco* prediction of new functions and functional links for well-known proteins. It was shown to be effective in application to missing links in biochemical pathways [7], [8], However, genomic functional coupling paradigm may also change our understanding of the landscape of biological functional interactions on a larger scale opening new approaches for modulation of cellular and organismal characteristics.
